# “Off with the Old”: Mindfulness Practice Improves Backward Inhibition

**DOI:** 10.3389/fpsyg.2012.00618

**Published:** 2013-01-11

**Authors:** Jonathan Greenberg, Keren Reiner, Nachshon Meiran

**Affiliations:** ^1^Department of Psychology, Zlotowski Center for Neuroscience, Ben-Gurion University of the NegevBeer-Sheva, Israel

**Keywords:** mindfulness, backward inhibition, competitor rule suppression, task switching, mental set

## Abstract

Mindfulness practice has been linked to reduced depressive rumination and described as involving inhibition of information that has been relevant in the past and is no longer relevant in the present moment. Backward inhibition (BI) is considered to be one of the purest measures of task set inhibition, and impaired BI has been linked to depressive rumination. BI was contrasted with Competitor Rule Suppression (CRS), which is another phenomenon observed in task switching, yet one which involves episodic memory tagging of information that is currently conflicting rather than active inhibition. Although similar at baseline level, a randomly assigned group (*n* = 38) who underwent an eight session mindfulness training program exhibited improved BI but not CRS compared to a waiting list group (*n* = 38). Findings indicate that mindfulness improves the specific component of task set inhibition, which has previously been linked to reduced rumination. Implications regarding the potential role of task set inhibition in mediating between mindfulness and reduced rumination, as well as the role of mindfulness in “being in the present moment” are discussed.

## Introduction

In a changing environment, the ability to inhibit thoughts and actions which are no longer relevant is crucial for our everyday functioning and well being. Impairments in such inhibition may result in perseveration, being “stuck” in a certain way of thinking, and responding inappropriately rather than adhering to the current situation demands (Mayr and Keele, 2000). Responding to a new situation or task requires deactivation of mindsets adopted in the recent past in favor of the currently relevant mindsets (see Koch et al., 2010).

In this paper we propose that mindfulness practice is a method which may promote such inhibition. Mindfulness has been defined as “paying attention in a particular way: on purpose, in the present moment and non-judgmentally” (Kabat-Zinn, 1994, p. 4). Although various definitions and conceptualizations have been proposed for mindfulness (see Chambers et al., 2009; Chiesa and Malinowski, 2011; Chiesa and Serretti, 2011; Grossman and Van Dam, 2011, for discussion), there is relative agreement that mindfulness involves both self regulation of attention to the present moment, and an orientation of openness and acceptance toward it. Such attendance to the present moment seems likely to involve inhibition of past thoughts and representations (Bishop et al., 2004).

Inhibition has been proposed to be one of the three cognitive processes termed “executive functions,” with the other two being mental set shifting and information updating and monitoring (Miyake et al., 2000). These functions are considered to enable the exertion of cognitive control and flexible adaptation, typically in novel or otherwise highly demanding contexts. Participants undergoing mindfulness training exhibited equivalent performance to control groups in a mental set shifting paradigm (Anderson et al., 2007) and in an internal switching paradigm requiring both set shifting and working memory updating (Chambers et al., 2008), yet outperformed controls on a multiple perspective images task requiring participants to switch between visual perspectives (Hodgins and Adair, 2010). Studies which have addressed the relation between mindfulness and various processes which may involve the component of inhibition have yielded an even more mixed pattern of results (see Chiesa and Serretti, 2011). People undergoing mindfulness training have been shown to exhibit superior performance relative to control groups on the Hayling task, requiring participants to complete sentences with unrelated and nonsensical words (Heeren et al., 2009), verbal fluency (Heeren et al., 2009; Zeidan et al., 2010), tasks requiring participants to respond to an arrow pointing to either the same or opposite direction as flanking arrows (Hodgins and Adair, 2010, in long term meditators; Tang et al., 2007), and on the Einstellung water jar task, which examines the degree to which participants are able to identify and utilize simple and obvious solutions to problems following repeated experience with a long and complex solving method (Greenberg et al., 2012). Conversely, no differences were found between mindfulness practitioners and control groups on other measures which may involve inhibitory processes, such as the GoStop paradigm (Heeren et al., 2009), and the continuous performance task (Cusens et al., 2010), both of which require participants to withhold their response whenever a certain signal appears. Additionally, findings regarding the effect of mindfulness on Stroop interference have yielded inconsistent results (see Anderson et al., 2007; Chan and Woollacott, 2007, in long term meditators; Josefsson and Broberg, 2011, in long term meditators; van den Hurk et al., 2010, in long term meditators; Wenk-Sormaz, 2005).

The results described above make it difficult to draw clear conclusions regarding the relation between mindfulness and inhibition. One reason for this difficulty is the clear inconsistency of findings. More importantly, however, is that most if not all of the above findings may be explained by various non-inhibitory accounts such as episodic memory retrieval, persistent activation, resolution of conflict between various possible routes, and rule guided algorithmic processing (see MacLeod et al., 2003; Neill, 2007; Koch et al., 2010). For example, while the Stroop effect has been linked to cognitive control and inhibition (Roelofs, 2003) it has also been described as *the* paradigmatic example for automaticity (Tzelgov, 1997).

In this paper, we focus on the relation between mindfulness practice and two measures related to task set suppression, which are both identified within the same task switching paradigm. An important advantage of the fact that the two measures are taken from the same paradigm is that whatever differences are found between them could not be attributed to superficial differences between paradigms. Critically, while one of the measures taps active online inhibition of information that has been relevant in the past but is no longer relevant, the other measure taps (mostly) episodic tagging of *currently* competing information. We will hereby briefly describe each of these measures.

*Backward Inhibition* (BI, Mayr and Keele, 2000), also referred to as *n-2 repetition cost* (Koch et al., 2010), can only be assessed in paradigms involving switching between three or more tasks. When switching from Task A to Task B, Task A is thought to be inhibited. In this case, Task A-related information was relevant in the past, but now it is no longer relevant since Task B became relevant instead. Therefore, switching back to task A following its recent inhibition (A→B→A task sequence) should be more difficult than switching to task A without its recent inhibition (e.g., following a C→B→A task sequence) because of the residual active inhibition of Task A. The relative difference between sequences ABA and CBA in reaction time and/or accuracy is thought to reflect the degree to which Task Set A was actively inhibited in the previous trial (Mayr and Keele, 2000; Koch et al., 2010). As mentioned above, to date, the only account of the *n*-2 repetition cost is based on inhibition (Koch et al., 2010). Therefore, examining the influence of mindfulness practice on BI is especially revealing in this respect. Moreover, BI indexes the inhibition of things that have been relevant in the recent past, presumably making it possible to focus on the current task. Thus, aside from being a pure measure of inhibition, unlike most of the other measures that have been used thus far, the type of inhibition tapped by BI (of previously relevant information) is especially relevant to mindfulness, due to its emphasis on focusing on the present moment.

Although, to our knowledge, the relation between BI and mindfulness practice has not been examined, we posit that it is likely that the two are positively related. Our reasoning is twofold. First, like BI, mindfulness has been claimed to involve inhibition of thoughts and representations that were relevant in the past but are no longer relevant (Bishop et al., 2004). Impairments in task set inhibition (i.e., using no longer relevant task sets) may be taken as a form of being “stuck in the past” rather than attending to the present moment. A second reason for this assumed relation is that both mindfulness (Ramel et al., 2004; Kingston et al., 2007; Chambers et al., 2008; Heeren et al., 2009; Michalak et al., 2011; Campbell et al., 2012) and BI (Whitmer and Banich, 2007; Whitmer and Gotlib, 2012) have been linked to reductions in depressive rumination, thereby further implying a possible link between the two phenomena.

The second measure which we examined is *Competitor Rule Suppression* (CRS; Meiran et al., 2010, 2011b). In order to explain CRS we must first explain the concept of a competing rule. A competing rule is a rule that generates a response that competes with the correct response. Take for example an experimental trial in which the relevant task rule is Gender (requiring a male-female decision) and indicating the right key as the correct response. Any task rule (such as Hair Color) that would implicate the left key as the correct response would thus be defined as “competing rule.” Suppression, according to this postulation, is evident if the previously competing rule becomes the relevant one in the following trial. Thus, CRS is computed by comparing performance (RT and accuracy) on trials in which the current task rule was a competing task rule in the previous trial with all other trial types, in which the current task rule was not the competing one in the previous trial (including trials in which another rule was competing). A recent study by Hsieh et al. (2012) demonstrated that CRS is not primarily accounted for by residual active inhibition of competing rules. Instead, it seems to mostly reflect the tagging of currently conflicting rules as rules that should not be processed when storing the processing episode in memory. When the tagged rules become relevant in the following trial, they are retrieved with the “do-not-process” tag, thereby impairing performance. Thus, BI and CRS differ in two aspects. While BI reflects *residual active inhibition* that took place *in the previous trial*, CRS reflects “do-not-process” *tagging* of *currently competing* rules. Thus, while Both BI and CRS may be seen as phenomena promoting attendance to the present moment, they do so in a different way, with the former involving active inhibition, which has previously been linked to mindfulness (e.g., Bishop et al., 2004), and the latter involving episodic memory tagging of currently competing information.

The aim of the current paper was to examine whether mindfulness specifically improves inhibition of no longer relevant mindsets. If so, participants undergoing a mindfulness training program should exhibit improved BI. If mindfulness practice involves tagging of past information as irrelevant in episodic memory rather than its inhibition, participants undergoing a mindfulness training program should exhibit improved CRS but not improved BI. To examine this issue, we compared two randomly assigned groups of non-meditators: a group that underwent eight sessions of mindfulness training (“mindfulness”), and a “waiting list” group. Groups were compared on a measure of BI and CRS both before and after mindfulness training of the mindfulness group.

## Materials and Methods

### Participants

The participants and experimental design were those used by Greenberg et al. (2012). Seventy six individuals with no former meditation experience were recruited via poster ads hung around Ben-Gurion University campus and email ads sent to all university students, offering a free mindfulness program for those participating in two experimental sessions. The program was due to start in two possible dates several months apart. Exclusion criteria included people with learning disabilities and non-native Hebrew speakers, as well as people with previous meditation background.

Following the first experimental session, participants were randomly assigned to a Mindfulness meditation group (*N* = 38) intended to participate in the first program and a waiting list (control) group (*N* = 38) intended to participate in the second program. No significant differences were found between groups in Age [*M* = 25.45, SD = 2.56 for meditators, *M* = 26, SD = 2.5 for controls, *t*(74) = 0.95, *ns*], gender (13 male meditators, 15 male controls, *p* = 0.41, Fisher’s exact test), nor in academic abilities as measured by self reported Psychometric Entrance Test (PET) scores, the Israeli equivalent of the SAT scores [*M* = 662.97, SD = 62.22 for meditators, *M* = 672.66, SD = 57.37 for controls, *t*(74) = 0.70, *ns*]. The two groups were additionally equivalent in ratings of happiness, sadness, and general mood [maximal *t*(73) = 0.69, *ns*]. As measured by asking participants to rate their current degree of happiness, sadness, and general mood on a 1–9 Likert scale. Emotion rating data were available for all but one participant later assigned to the mindfulness group. The experiment had received approval from the psychology department’s ethics committee in Ben-Gurion University.

### Measures

The CRS paradigms were similar to those used by Meiran et al. (2010, 2011b). These paradigms have been demonstrated to produce both reliable BI and CRS effects. Two similarly structured measures were administered before and after the mindfulness group underwent the mindfulness program^1^. The experiments were run on Pentium 4 computers with 17″ (43.18-cm) monitors. The procedures were programmed in E-Prime 2 (Psychological Software Tools, Inc., 2005).

#### “Vertical Boxes” paradigm (pre-test)

**Figure 1:**
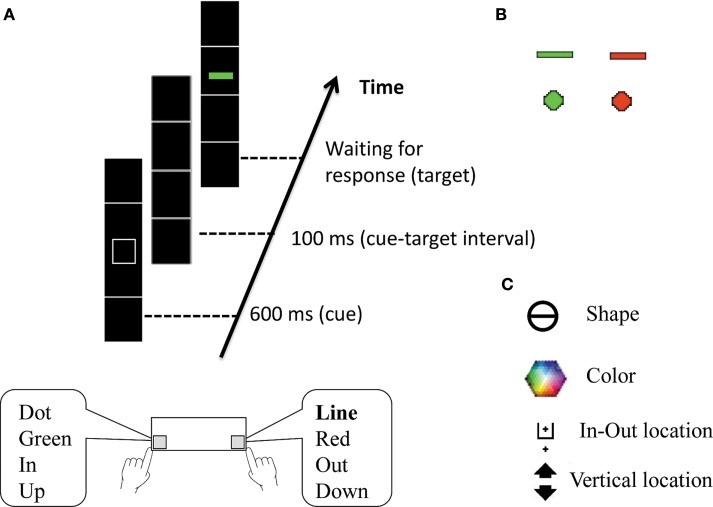
**Illustration of the Vertical Boxes paradigm, based on Meiran et al. (2011a)**. **(A)** Schematic representation of the events in a trial, including cue presentation, followed by target presentation; (**B)** the four target objects; (**C)** the cues for the four tasks.

This paradigm was used to assess equivalence between groups in BI at baseline level, in Session 1, prior to the administration of the mindfulness program. We had used a shorter and slightly modified version of the paradigm described in detail by Meiran et al. (2011b). Each trial started with a presentation of a black screen for 500 ms, which served as a response-cue interval. A vertical array of four boxes then appeared for 600 ms, with one of four possible task related cues in its center, indicating which of four tasks is to be performed. Following a screen displaying only the array of boxes for 100 ms, a target stimulus (in the form of either a dot or line colored in either red or green) then appeared in one of the four boxes (see Figure 1). The four tasks were an “in-out” task in which participants had to judge whether the target stimulus appeared in the inner two boxes (“in”) or in the outer (top or bottom) boxes (“out”), as well as tasks requiring judgment of color (red vs. green), shape (dot vs. line), and vertical location of the target (i.e., upper two boxes vs. lower two boxes). Participants had to press the “A” key (on the left) if the target was “in,” green, a dot, or “up,” and the “L” key (on the right) if the target was “out,” red, a line, or “down,” respectively for the four tasks. A 400 Hz beep sounded in case of an error in response. Due to the randomized controlled study design, we decided not to counterbalance keys, in order to assure standardization as much as possible.

Participants received instructions introducing the tasks and stimuli, and were instructed to use their index fingers, and respond as quickly and accurately as possible. They then performed one single-task block of 50 trials for each of the four tasks (in-out, color, shape, and vertical location). Following this block, participants performed one practice block of 64 trials in which the four tasks were randomly mixed, followed by seven experimental blocks of 64 trials. At the end of each block, participants were instructed to take a short break, stretch or walk around for a short while, and continue when ready. One modification from the paradigm used by Meiran et al. (2011b) was practicing each task separately at the beginning of the experiment rather than gradually adding tasks. This ensured equal experience with each task prior to mixing the four tasks together. A second modification was a reduction in the number of experimental blocks (7 instead of 18) in order to shorten the length of the experiment. As mentioned above, keys were not counterbalanced in order to increase standardization. A final modification was in the cue for the Shape task, which was modified from a square to a circle with a horizontal line running through it. Since the targets were either a circular dot or a horizontal line, the cue was altered to better represent the targets.

#### “Faces” paradigm (post-test)

This paradigm was used to assess differences between groups in Session 2, after the mindfulness group completed the mindfulness program. This paradigm is a shorter and slightly modified version of the one described in detail by Meiran et al. (2010). Modifications include practicing each task separately at the beginning of the experiment, and using 7 rather than 18 experimental blocks, for the reasons described above. The paradigm was identical in block structure to the “vertical boxes” paradigm used in the first experimental session with the exception that the cue was kept onscreen until a response was given. However, the Faces paradigm involved different stimuli, response keys, and mostly different tasks.

**Figure 2:**
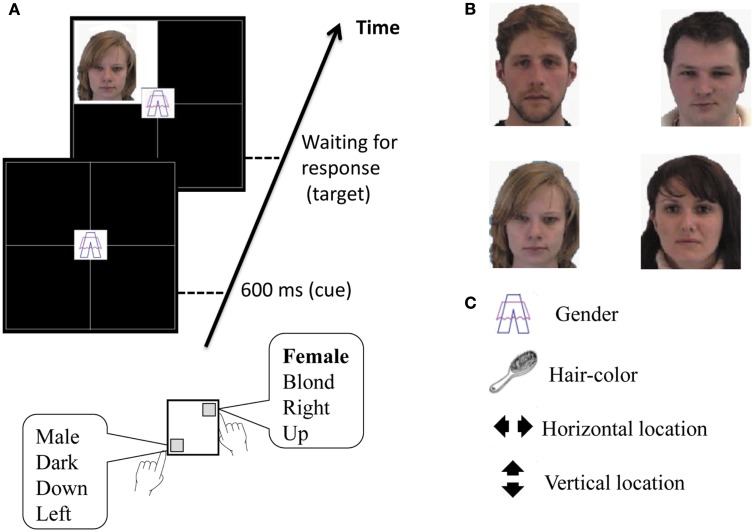
**Illustration of the Faces paradigm, based on Meiran et al. (2010)**. **(A)** Schematic representation of the events in a trial, including cue presentation, followed by target presentation; **(B)** the four target objects; **(C)** the cues for the four tasks.

A 2 × 2 array of boxes appeared onscreen with one of four possible task related cues in its center, indicating which of the four tasks is to be performed. A target stimulus in the form of one of four possible faces of young adults, two blonde and two dark-haired, then appeared in one of the four boxes. The four tasks were a horizontal location task, a hair color task, a gender task, and a vertical location task (see Figure 2). Participants had to press the “V” key (on the lower-left side) if the face target appeared in the left column of boxes, if the hair was dark, if target was a face of a male, and if it appeared in the bottom row of boxes, and press the “U” key (on the upper-right side) if the face appeared on the right column, if it was a face of a female, the hair was blonde, and if it appeared in the top row of boxes, respectively for the four tasks. A 400 Hz beep sounded in case of an error in response. As in the “vertical boxes” paradigm, following instructions participants performed one single-task block of 50 trials for each of the four tasks (horizontal location, hair color, gender, and vertical location), then performed a practice mixed-task block of 64 trials, followed by seven experimental similar blocks. Blocks were separated by an instruction to take a short break.

#### Self reported emotion ratings

Participants were asked to rate their degree of happiness, sadness, and general emotional mood on a Likert scale ranging from 1 to 9 in order to control for performance differences which may be accounted for by mood.

### Mindfulness program

The program, based on Mindfulness Based Cognitive Therapy (MBCT) program (Segal et al., 2002) with adaptations to include handling everyday stress rather than only depression, is described in detail by Greenberg et al. (2012). The program consisted of eight meetings, held over 6 weeks. Seven meetings were 2 h long, and one was a half day “retreat” which took place at the end of the fifth week. The instructor was one of the authors (KR), a trained mindfulness based meditation instructor having over 10 years of personal meditation experience. The instructor, who was blind to the hypothesis of the experiment, guided participants through mindfulness meditation practices such as breathing meditation, body scan, open awareness meditation, walking meditation, and compassion meditation, as well as through various awareness exercises, stories, and group discussions. The program required at least 20 min of daily home formal meditation practice. Participants received a daily email notification directing them to an online diary for filling a report of their daily home practice. Mean session attendance was 80%, and mean total practice time in the program was 19.65 h per participant for those included in the main analysis.

### Design

A mixed design was used. Group (mindfulness vs. waiting list) was the independent (between subject) variable, and the dependent (within subject) variables were BI (i.e., DBA and CBA vs. ABA trials) and CRS (i.e., trials in which the relevant rule was the interfering rule in Trial *n*-1 vs. trials in which the relevant rule did not interfere in Trial *n*-1).

### Procedure

Participants signed an informed consent form at the beginning of each of the two experimental sessions. In Session 1, participants performed the “vertical boxes” paradigm. They were then randomly assigned to a waiting list and a mindfulness group, using the “random” function in Python programming language. In Session 2, which took place near the end of the mindfulness program, participants performed the “faces” paradigm. Toward the end of each session participants rated their mood using the self reported emotion rating.

## Results

Since both BI and CRS occur only in the setting of switching between tasks, we report here data from the seven experimental mixed-task blocks. Trials in which RT’s were lower than 100 ms (anticipatory errors) and higher than 3500 ms (outliers) were eliminated (together 1.1% of the trials), as were the two trials following an error or the two trials from the beginning of the block were eliminated, as required to assess BI (see Mayr and Keele, 2000; Meiran et al., 2010, 2011a). Since different paradigms with different stimuli, response keys, and tasks were used in the two experimental sessions, we did not analyze the results of the two paradigms in a single analysis (with pre-post serving as a repeated measures variable) and results from the two sessions are hereby reported separately.

### “Vertical boxes” paradigm (pre-training)

A three way analysis of variance (ANOVA) was conducted, with Group (waiting list vs. mindfulness), BI (i.e., DBA and CBA vs. ABA trials), and CRS (trials in which the relevant rule was the interfering rule in Trial *n*-1 vs. trials in which the relevant rule did not interfere in Trial *n*-1) as the independent variables, and RT as the dependent variable. A significant main effect was found for CRS, in which participants were overall slower on trials in which the relevant rule was the interfering rule in the previous trial [*F*(1, 74) = 14.93, *p* < 0.001]. A just-significant interaction was found between Group, BI, and CRS [*F*(1, 74) = 3.98, *p* = 0.05]. Planned comparisons indicate that participants who were later assigned to the mindfulness group exhibited a non-significant trend of a greater BI effect in the non-CRS condition [*F*(1, 74) = 2.33, *ns*], whereas those later assigned to the waiting list group exhibited a similar trend in the CRS condition [*F*(1, 74) = 3.03, *ns*]. A significant main effect was also found for BI [*F*(1, 74) = 24.38, *p* < 0.001], indicating that participants were overall slower to respond on BI trials than on control trials (i.e., ABA task sequence vs. CBA or DBA task sequence). A similar ANOVA was conducted with Error Rate as the dependent variable. A significant main effect was found for CRS [*F*(1, 74) = 11.88, *p* < 0.001], indicating that in addition to being slower, participants were also less accurate in CRS trials. A marginally significant main effect was found for BI [*F*(1, 74) = 3.91, *p* = 0.05], indicating that overall, participants made more errors in trials which did *not* require BI (i.e., CBA or DBA task sequence) compared to trials which did require BI (i.e., ABA task sequence). This indicates a tradeoff between speed and accuracy for both groups, in which participants were slower but more accurate in BI trials. No other effects approached significance.

**Figure 3:**
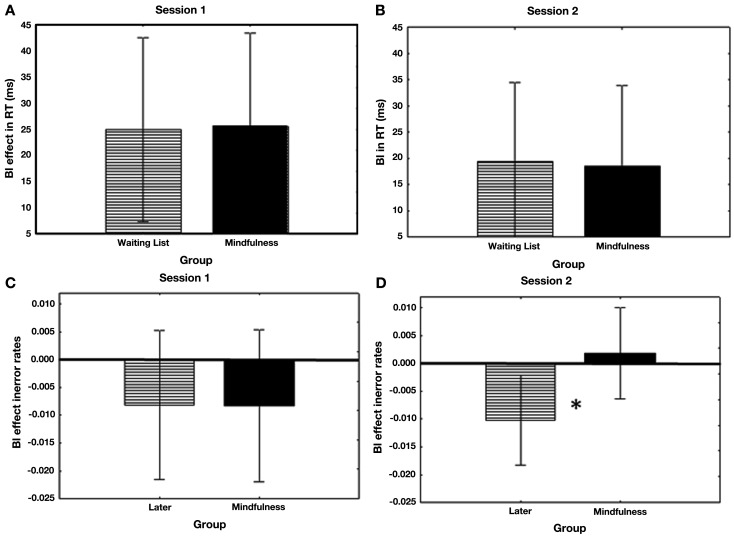
**BI RT’s and error rates at baseline level and after the mindfulness program**. Backward Inhibition (ABA task sequence trials-CBA task sequence trials) according to Group in **(A)** RT in Session 1, **(B)** Error Rate in Session 1, **(C)** RT in Session 2, and **(D)** Error Rate in Session 2. The positive BI effect in RT **(A)** and negative BI effect in Error Rate **(B)** suggest a speed-accuracy tradeoff, with no reliable BI effect at baseline level. Although the waiting list group exhibited a similar trend and tradeoff at Session 2, the mindfulness group exhibited a positive BI effect in RT **(C)** *and* a positive BI trend in Error rate **(D)** thereby demonstrating a reliable BI post-intervention. The asterisk indicates that the mindfulness group exhibited a significantly greater BI effect in error rates than controls **(D)**.

Importantly, no significant differences were found between groups in BI nor CRS, which was true for both RT and error rates [maximal *F*(1, 74) = 0.98, *ns*; means are listed in Table 1]. This lack of differences in BI and CRS at baseline level remained, both in RTs and Error Rates [maximal *F*(1, 63) = 1.47, *ns*] after excluding participants who were excluded from analysis of the “faces” paradigm (see Figure 3). Thus, groups were statistically equivalent in BI and CRS at baseline level. Although participants in both groups seemingly exhibited a BI effect in RT, the significant reversed BI effect found in error rates indicates a speed-accuracy tradeoff. This tradeoff seems to reflect a bias toward a slower and more accurate performance strategy (see Wagenmakers et al., 2007) and suggests that there was no evidence for a reliable BI in either group at baseline level.

**Table 1:** **BI and CRS means and SD’s (in parenthesis) at baseline level and after the mindfulness program**.

		BI RT		BI error rate		CRS RT		CRS error rate	
		Non-BI	BI	Non-BI	BI	Non-CRS	CRS	Non-CRS	CRS
Mindfulness group	Session 1	825 (37)	853 (37)	0.053 (0.011)	0.047 (0.007)	823 (36)	855 (39)	0.046 (0.009)	0.054 (0.009)
	Session 2	622 (33)	640 (32)	0.040 (0.009)	0.042 (0.009)	616 (31)	645 (35)	0.038 (0.009)	0.045 (0.009)
Waiting list	Session 1	830 (37)	856 (37)	0.053 (0.011)	0.043 (0.006)	833 (36)	853 (39)	0.042 (0.009)	0.055 (0.009)
	Session 2	660 (33)	679 (32)	0.0386 (0.009)	0.028 (0.009)	662 (30)	677 (35)	0.031 (0.009)	0.036 (0.009)

### “Faces” paradigm (post-training)

Data were unavailable from four participants from the waiting list group and two from the mindfulness group who chose not to participate in Session 2. Data were excluded from four other participants from the mindfulness group who attended less than four program meetings, and one participant from the waiting list group who had her dominant hand in a cast following an injury between the two experimental sessions. The main analysis was therefore conducted on 32 participants from the mindfulness groups and 33 from the waiting list group.

A three way ANOVA was conducted on RT with the same independent variables as above. A main effect was found for CRS [*F*(1, 63) = 12.80, *p* < 0.001], in which participants were overall slower on CRS trials. No significant interaction was found between CRS and Group [*F*(1, 63) = 1.12, *ns*]. A main effect was also found for BI [*F*(1, 63) = 12.31, *p* < 0.001], indicating that participants were overall slower on BI trials. The interaction of Group and BI was non-significant [*F*(1, 63) = 0.005, *ns*], indicating that both groups were equally slower on trials which required BI (see Figure 3; means are listed in Table 1). No other effects approached significance.

A similar ANOVA was conducted with Error Rate as the dependent variable. A significant main effect was found for CRS [*F*(1, 63) = 6.51, *p* < 0.05], yet it was statistically comparable for both groups, as indicated by the non-significant interaction [*F*(1, 63) = 0.65, *ns*]. No significant main effect was found for BI [*F*(1, 63) = 2.12, *ns*], yet the groups differed in BI, as indicated by the significant Group X BI interaction [*F*(1, 63) = 4.39, *p* < 0.05; see Figure 3]. *Post* *hoc* contrast comparisons reveal that while the waiting list group had a higher error rate on control trials [CBA and DBA; *F*(1, 63) = 6.41, *p* = 0.01], the mindfulness group exhibited an opposite (non-significant) trend (see Figure 3; see Table 1 for means). This differential performance of the two groups in BI error rates remained significant after adding BI and CRS error rates from the “Vertical Boxes” paradigm as covariates to the analysis in an Analysis of Covariance [ANCOVA; *F*(1, 61) = 4.06, *p* < 0.05]. Although no differences between groups were found in self reported sadness, happiness, or general mood ratings following the mindfulness program [maximal *t*(63) = 0.99, *ns*], these ratings have been added as covariates in a separate ANCOVA examining a similar error rate analysis. Differences between groups in BI error rates remained significant in this case as well [*F*(1, 60) = 4.31, *p* < 0.05].

In order to examine whether the mindfulness program altered the way participants reacted to making an error or the tone played when an error was made, post error slowing was calculated by subtracting RT’s of trial in which the previous response was correct from trials in which the previous response was an error. A *t*-test was conducted comparing the groups in this measure indicated that the two groups were equivalent in post error slowing [*t*(63) = 0.05, *ns*], and therefore did not react differently to making in error or the tone played.

In sum, with regards to CRS, both groups exhibited a reliable effect in the two experimental sessions, yet did not differ between them. With regards to BI, the waiting list group exhibited a similar BI pattern in both experimental sessions, and adopted a strategy of slower but more accurate performance on BI trials, with no indication of a reliable BI effect. In contrast, the mindfulness group exhibited a different pattern of BI results in the two experimental sessions. While their performance was similar to that of the waiting list group at baseline level (i.e., BI effect in RT with a reversed BI effect in error rates, which renders the RT-BI effects unreliable), after the program they exhibited a reliable RT-BI effect with a similar trend in error rates, without an indication of speed-accuracy tradeoff. This indicates that mindfulness meditation resulted in a clear and reliable RT-BI effect as opposed to an RT-BI effect resulting from a speed-accuracy tradeoff (rather than processing efficiency) that characterized the control group.

## Discussion

Mindfulness practice is said to be characterized by inhibition of information not relevant to the present moment (e.g., Bishop et al., 2004), and has been shown to reduce depressive rumination (Ramel et al., 2004; Kingston et al., 2007; Chambers et al., 2008; Heeren et al., 2009; Michalak et al., 2011; Campbell et al., 2012). BI is considered to be one of the “purest” measures of task set inhibition (Koch et al., 2010), and impairments in it have been linked to depressive rumination (Whitmer and Banich, 2007; Whitmer and Gotlib, 2012). Given the fact that switching between tasks is a way to operationalize switching between mindsets (Meiran, 2010), BI indicates the ability to inhibit the mindset that had been adopted in the recent past in favor of the currently relevant mindset. We therefore examined the hypothesis that mindfulness would improve BI. Additionally, since many of the observed differences between mindfulness practitioners and controls in measures assumed to be related to inhibition (see Introduction) may be explained by non-inhibitory accounts such as episodic memory retrieval (see MacLeod et al., 2003; Koch et al., 2010) we also examined group differences in CRS, which primarily taps the process in which competing rules are tagged in episodic memory.

The two groups exhibited similar BI trends in Session 1. In fact, both groups showed BI in RT but showed a significant reversed trend in Error Rate. Thus, there was no clear indication for a reliable BI effect in Session 1. Rather, the trend indicated a speed-accuracy tradeoff. In Session 2, the control group continued to show this trend of tradeoff. However, the mindfulness group showed a reliable BI in RT and a similar trend in errors, indicating a reliable BI effect without speed-accuracy tradeoff. Thus, we conclude that mindfulness training improves BI.

While BI results differed between groups, CRS results did not. This differential effect of the mindfulness program may shed some light as to how mindfulness may promote attendance to the present moment. Both BI and CRS may be seen as phenomena promoting attendance to the present moment, yet do so via differential mechanisms. BI seems to conduce to attending to the present moment by the active inhibition of information which was recently relevant but is no longer so. With past information inhibited, one may more easily notice and attend whatever currently arises. In contrast, CRS seems to conduce to attending to the present moment by tagging in episodic memory of information which is currently competing as “not to be responded to.” Results of the study suggest that mindfulness improves the specific component of inhibition of no longer relevant information rather than episodic memory tagging of competing information.

Such improved inhibitory ability among mindfulness practitioners seems of relevance to the use of mindfulness based interventions in treatment of various clinical conditions, due to the central role played by inhibition in many of these conditions such as obsessive compulsive disorder (Enright and Beech, 1993), schizophrenia (Nestor and O’Donnell, 1998), and attention deficit/hyperactivity disorder (Barkley, 1997; Nigg, 2001). With regards to cognitive aging, BI was found to be unimpaired in old age (Mayr, 2001). The present findings provide support to the notion that neurocognitive mechanisms involved in mindfulness would not limit its efficacy in old age. This receives additional support from findings that mindfulness is often found to be an effective intervention in the elderly (see Lindberg, 2005; McBee, 2008). An additional contribution of the current results involves the relation between mindfulness and rumination. Rumination has been found to be one of the major risk factors in depression, and typically consists of repeated thinking about one’s negative symptoms, their causes, and implications (Nolen-Hoeksema, 1991). One of primary aims of MBCT is to become aware of such negative thinking patterns, and disengage from them, thereby reducing the risk for a relapse in depression (Segal et al., 2002). Albeit the currently available evidence indicating that mindfulness practice is related to reduced rumination (Ramel et al., 2004; Kingston et al., 2007; Chambers et al., 2008; Heeren et al., 2009; Michalak et al., 2011; Campbell et al., 2012), little if any empirical evidence exists regarding *how* mindfulness does so. Impairments to BI are assessed using different methods, and typically observed in shorter time periods (Mayr and Keele, 2000) compared to rumination. Nevertheless, both BI and rumination involve perseveration of a particular mindset or way of thinking, and a difficulty in moving on from information which is no longer relevant. Both phenomena also often result in maladaptive reasoning or responding due to such difficulty in inhibition of previous mindsets (Nolen-Hoeksema, 1991; Mayr and Keele, 2000). Thus, despite some differences between the two phenomena, the similarities described above suggest that they may have a common base. By coupling the current finding that mindfulness improves BI with previous findings suggesting that impairments to BI are associated with trait depressive rumination (Whitmer and Banich, 2007; Whitmer and Gotlib, 2012), we tentatively suggest a mechanism in which mindfulness may reduce rumination via increased inhibition of no longer relevant information and task sets.

The rationale for our hypothesis that mindfulness may improve BI was largely based on the fact that inhibition of no longer relevant information may conduce to attending the present moment. One may suggest that the finding that mindfulness training improves BI may be taken as evidence to the contrary, that mindfulness decreases rather than increases present moment awareness. This is because BI entails lingering of inhibition from previous trials and, in a way, reflects some “carrying over” of the past (i.e., the carrying over of inhibition). We posit, however, that such inhibition conduces much more than it impairs attendance to the present moment, as the lack of such inhibition often results in perseveration and getting “stuck” in no longer relevant mental sets (Mayr and Keele, 2000). An additional point which we wish mention is that since both groups were assessed prior to the mindfulness program, we cannot rule out the possibility that mindfulness improves BI only after such a pre-test, although this seems highly unlikely.

The speed-accuracy tradeoff observed in the current study was not significantly observed by Meiran et al. (2010), who have used a longer version of the Faces paradigm. One may interpret such difference between studies in speed-accuracy tradeoff as potential threats to validity and or reliability of BI findings in our study. However, both studies have found significant and similar baseline BI effects in RT (roughly 27 ms) thus providing evidence for validity in BI measurement in the current study. Moreover, Meiran et al. found no BI effect in error rates (less than 0.001 in proportion of errors, *ns*) meaning that approximately half of their participants exhibited the same pattern of negative BI performance in error rates as displayed by the sample in the current study. With regards to the reliability of BI measurement, the fact that both groups in the current study exhibited a qualitatively similar tradeoff at baseline level, and that this finding was repeated by the control group at Session 2 indicates that this finding in the absence of mindfulness practice is reliable. In other words, our sample was slightly different in its characteristics than that of Meiran et al. (2010), yet the sample was successfully divided by the random assignment into two equivalent groups and the mindfulness intervention significantly altered the BI pattern in the experimental group.

Comparing a randomly assigned mindfulness group with a waiting list group may be taken as a limitation of the current study, as it only enables us to attribute findings to the mindfulness intervention as a whole, rather than to specific components within the intervention, as may have made possible by using an active control group. In addition, although the relation between BI and depressive rumination has been previously established (Whitmer and Banich, 2007; Whitmer and Gotlib, 2012), we did not measure rumination in the current study. Future studies may compare mindfulness with other intervention programs to clarify the role of specific components in mindfulness interventions, and may also extend the use of this measure in various forms of psychopathology in order to broaden our understanding of the efficacy of mindfulness practice in treatment of these conditions.

## Conflict of Interest Statement

The authors declare that the research was conducted in the absence of any commercial or financial relationships that could be construed as a potential conflict of interest.

## Footnotes

^1^Since participants were randomly assigned to groups, participants may be examined using only a post-test. Nevertheless, we had still decided to examine BI and CRS at baseline level to further insure lack of differences at baseline level. Although similarly structured, the two paradigms are analyzed separately since they differed in stimuli and tasks (which was chosen in order to avoid practice effects).
